# Dyeing Non-Recyclable Polyethylene Plastic with Photoacid Phycocyanobilin from Spirulina Algae: Ultrafast Photoluminescence Studies

**DOI:** 10.3390/polym14224811

**Published:** 2022-11-09

**Authors:** Maryam Alhefeiti, Falguni Chandra, Ravindra Kumar Gupta, Na’il Saleh

**Affiliations:** 1 Department of Chemistry, College of Science, United Arab Emirates University, Al Ain P.O. Box 15551, United Arab Emirates; 2 King Abdullah Institute for Nanotechnology, King Saud University, Riyadh 11451, Saudi Arabia

**Keywords:** low-density polyethylene, phycocyanobilin, time-resolved fluorescence, ionic transport

## Abstract

Despite the enormous environmental damage caused by plastic waste, it makes up over one-third of globally produced plastics. Polyethylene (PE) wastes have low recycling but high production rates. Towards the construction of ionic solar cells from PE, the present work describes the loading of a bioactive photoacid phycocyanobilin (PCB) dye from the pigment of Spirulina blue–green algae (as a natural resource) on low-density polyethylene (LDPE) plastic film. Dyeing was confirmed by X-ray photoelectron spectroscopy (XPS). Upon excitation of the Soret-band (400 nm), the photoluminescence (PL) spectra of PCB in neat solvents revealed two prominent emission peaks at 450–550 and 600–700 nm. The first band assigned to bilirubin-like (PCB_BR_) species predominated the spectral profile in the highly rigid solvent glycerol and upon loading 0.45 % (*w*/*w*) of the dye on plastic. The photoluminescence excitation (PLE) spectra of PCB for the second region (Q-band) at 672 nm in the same solvents confirmed the ground state heterogenicity previously associated with the presence of PCB_A_ (neutral), PCB_B_ (cationic), and PCB_C_ (anionic) conformers. Time-resolved photoluminescence (TRPL) measurements induced via excitation of all PCB species at 510 nm in methanol revealed three-lifetime components with τ_1_ = ~0.1 ns and τ_2_ = ~2 ns associated with PCB_BR_ species and τ_3_ = ~5 ns pertinent to the long-living photoproduct X*. Decay-associated spectra (DAS) analysis of the photoluminescence transient spectra of the final dyed films in the solid-state confirmed the improved generation of the long-living photoproduct as manifested in a significant increase in the PL intensity (~100-fold) and lifetime value (~90 ns) in the Q-region upon loading 6.92 % (*w*/*w*) of the dye on plastic. The photoproduct species were presumably assigned to the deprotonated PCB species, suggesting improved ionic mobility. The potential implementation of the PCB-sensitized PE solid wastes for the fabrication of ionic solar cells is discussed.

## 1. Introduction

The conversion of sunlight to ionic current/power has become a popular topic over the past five years following the development of photovoltaic membranes made up of photoacid-linked polymers [1,2,3]. The discovery has expanded the applications of the classical semiconductor pn-junction electrode in the technology of solar cell devices. Photoacids are molecules that become more acidic in their excited state, leading to increased acidity and dissociation of protons (ions) [4]. The group of Ardo [1,2,3] presented various spectroscopic, photophysical, and theoretical evidence for the capabilities of these materials to convert light into ionic power through photo-driven transport of protons and hydroxides, leading to the discovery of what they called an ionic solar cell. For example, one electrolytic membrane was developed in their laboratory upon linking perfluoro sulfonic acid (PFSA) as the photoacid to ion-exchange polymers with the inherent capability of proton conduction such as Neosepta AHA [1] and Nafion [3], as well as to polyethylene terephthalate (PET) [2]. On a separate note, a stable Nafion-impregnated polyethylene (PE) membrane has also been fabricated for fuel cell application [5].

The most common plastic used in consumer plastics is high-density or low-density polyethylene (HDPE/LDPE), polypropylene (PP), polyvinyl chloride (PVC), and polystyrene (PS). In 2015, 68 million tons of PE was produced, making it the second-most produced plastic [6]. In the year 2050, the production rate for all plastic is expected to reach over 2000 million tons. PE is most used as a material for film applications, bags for groceries, bread, frozen food and garbage, beverage cups and squeeze bottles, food containers, wire cables in automobile applications, or as a material for medical applications and sterilization. It makes up over one-third of globally produced plastic [6]. Most PE plastic wastes are directed to landfills since they are non-biodegradable under natural environmental conditions. This increases the ecological problem as PE plastic wastes produce toxic additives such as lead and cadmium. They are considered to have poor UV resistance and are highly flammable [7]. This means PE is not the bad guy, but we have just made too much of it. PE recycling is very challenging. Mechanical, chemical, and biological depolymerization are the three most common plastic recycling strategies [6]. The complex and diverse polymeric structures of plastic materials make it challenging to degrade these materials using a single approach. Noticeably, PE is characterized by C-C single bonds [6]. Such long carbon-carbon backbones, hydrophobicity, and highly crystalline nature resist enzymatic attack. Moreover, chemical recycling requires high temperatures and a multi-step process to achieve a new polymer [6].

Most countries are now moving towards sustainability, and renewable energy and algae are great alternative energy sources that will revolutionize the industry, leading to advanced economies without contributing to global warming. Algae is receiving significant attention worldwide to harness its energy in the industrial and medical sectors, being a renewable, sustainable, and biodegradable source. Natural dyes and pigments gained attention over the past decades as eco-friendly replacements for industrial pigments. Algae is a naturally rich source of stains such as phycoerythrin, phycocyanin (PC), β-carotene, chlorophyll, fucoxanthin, and phycocyanobilin (PCB), which belong to tetrapyrrole (bilin) family [8,9].

The study of the photochemistry of PCB in solution gained recent attention [10]. The results in methanol concluded the existence in the ground state of three species of neutral PCB_A_, cationic PCB_B,_ and anionic PCB_C_. The first two luminescent species, PCB_A_* and PCB_B_*, decay in picoseconds, whereas PCB_C_* is not fluorescent. Furthermore, PCB_B_* species deprotonate in femtoseconds to a long-living photoproduct X*, which decays in nanoseconds. Here, our focus on PCB from phycocyanin protein-pigment in Spirulina blue–green algae is because we envisage it to display photoacidity properties. 

The long-term goal of the present investigation is to construct ionic solar cells based on linking PCB from the pigment of Spirulina algae on LDPE plastic films, specifically motivated by the fact that LDPE has low recycling rates, but high production rates and that Spirulina algae is a natural source PCB. Here, we link PCB onto an LDPE plastic film and discuss its potential use as a new photoacid-dye-sensitized bipolar ion-exchange membrane by analyzing its spectroscopy and photochemistry. 

## 2. Result and Discussion

### 2.1. Photophysics of PCB in Neat Solvents

**Figure 1:**
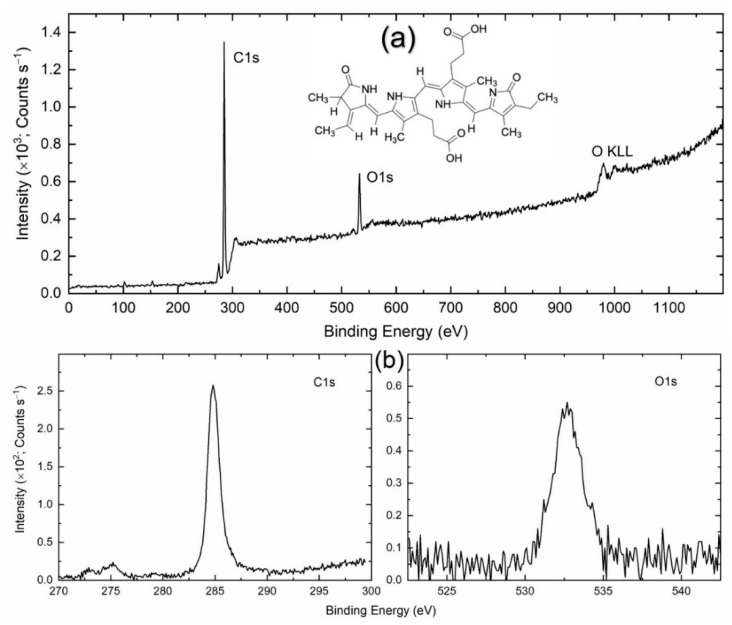
XPS spectra for (**a**) survey and (**b**) elements (C1s and O1s) of PCB-sensitized LDPE. The inset shows the chemical structure of PCB dye.

Previous optical measurements revealed weak, low-energy emission bands around 600–700 nm and high-energy emissions around 400–550 nm when PCB was excited in methanol at 360 nm (Soret-band) and 580 nm (Q-band) [10]. They concluded that the emission band at 400–550 nm is assigned to bilirubin-like chemical species (PCB_BR_), characterized by a strong S_0_–S_1_ transition. The structure of PCB, as illustrated in the inset of Figure 1, also permits its high flexibility to form three different types of conformers, PCB_A_ (neutral), PCB_B_ (cationic), and PCB_C_ (anionic), upon the rotation of the four pyrrole rings around their methine bridges. PCB_A_ and PCB_B_ absorb at 592 and 634 nm and emit at 636 and 665 nm, respectively. PCB_C_, in contrast, absorbs at 714 nm and has weak or no emission. The previous investigators also concluded from their transient absorption and fluorescence measurements of PCB in solution [9,10] that PCB_B_* and PCB_A_* species decay within a few picoseconds, and a long-living photoproduct X* generated in femtoseconds from PCB_B_* decay in nanoseconds. 

Table 1, Table S1, Figures S1 and S2 in the Supporting Information summarize spectral and photophysical measurements of PCB dye in neat solvents. In all solvents, except glycerol, the PL spectra display two emission bands in the 420–550 and 600–700 nm range when PCB is excited at 400 nm (Soret-band). The PLE spectra were measured when the emission wavelength was set at 672 nm (Q-band) to confirm the ground state heterogenicity previously associated with the presence of PCB_A_ (neutral), PCB_B_ (cationic), and PCB_C_ (anionic) conformers [10]. Moreover, the PL, PLE, and TRPL (excitation at 510 nm, see below) results confirm that the observed stokes shifts and average excited-state lifetimes (Experimental Method) of PCB do not correlate with all the solvent properties (polarity, polarizability, and hydrogen bonding) but with solvent viscosity. Noticeably, in the most viscous solvent glycerol, the average excited-state lifetimes (Table 1) dropped by half (viz. from ~2.0 versus ~1.0 ns) with significant concomitant evolution of the emission profile into one emission band at around 460 nm compared to all spectroscopic data in all other neat solvents in Table 1. Specifically, all the plots of stokes shifts (Δν) or excited-state lifetimes (*τ*) against solvent orientational polarizability (Δf), solvent polarity/polarizability factors (π*), solvent hydrogen bond accepting ability (β), or solvent hydrogen bond donating ability (α) gave scattered data with meaningless correlation coefficients. The results demonstrate the significant role of solvent rigidity in modulating the PCB’s photophysics. The rotation of the pyrrole moieties [10] in the structure of PCB may have become highly restricted in glycerol. This rigidity-effect explained the lack of emission peak in the Q region. It helps explain the photophysics of PCB linked to LDPE in the proceeding section. 

### 2.2. Interactions of PCB Dyes with LDPE Plastic Film: XPS Characterization

Figure S3a (Supporting Information) shows the XPS survey spectrum of PCB-sensitized Nafion. This is similar to the range of Nafion 117 [11]. The spectrum portrayed a strong peak for F1s, medium peaks for C1s and O1s, and a weak peak for S2p. It has no peak for N1s, an ingredient of the PCB. This is also reasonable. Theoretically, Nafion 117 contains 60% of F, 30.8% of C, 7.7% of O, and 1.5% of S [11,12]. We collected spectra for C1s, O1s, F1s, and S2p elements, as shown in Figure S3b. Table S2 and Figure S4 list the observed peak position for C1s, O1s, F1s, and S2p elements. This also includes the peak position of elements for pure Nafion and the associated functional group for comparison [11,12]. This showed a redshift in the peak position of elements, revealing the interaction of PCB molecules with Nafion polymer. We also observed a similar phenomenon between PCB molecules and LDPE, which is visualized in Figure 1. Figure 1a shows the XPS survey spectrum of PCB-sensitized LDPE. The spectrum depicted C1s and O1s peaks, corresponding to LDPE [13] and PCB [14], respectively. We, therefore, monitored C1s and O1s spectra, as shown in Figure 1b. The estimated peak positions for C1s and O1s of PCB-sensitized LDPE are listed in Table S3 and Figure S5, along with those of LDPE and PCB. This also demonstrated a redshift in peak position relative to pure LDPE [13], and high oxygen content [14] indicating an interaction between PCB and LDPE. 

### 2.3. Loading and PL Characterization

The loading of PCB guests on LDPE plastic film was quantified using UV−vis absorption spectroscopy (Experimental Section). A calibration curve was constructed from known concentrations of PCB dye in methanol (Figure S6 and Table S4 in the Supporting Information). Results revealed that the amount loaded on LDPE was 0.08, 0.44, and 6.92 (*w*/*w*) for each PCB concentration of 10, 100, and 1000 µM in methanol, respectively (Table S5 in the Supporting Information). 

### 2.4. PL and Time-Resolved PL Measurements

**Figure 2:**
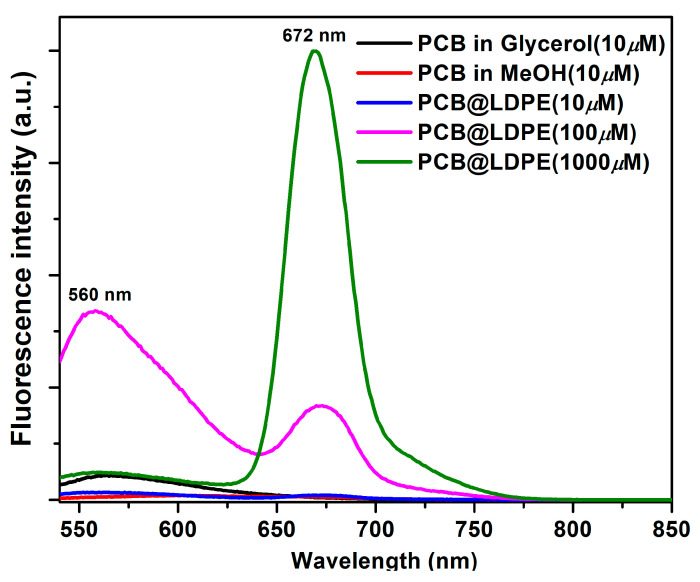
Evolution of the PL spectra of solid LDPE plastic film upon adding different concentrations of PCB dyes in methanol under vacuum at 298 K (solid samples were excited at 510 nm). The PL spectrum of PCB in glycerol was added for comparison.

Figure 2 illustrates the changes in the emission profile of PCB in methanol and when adsorbed on plastic at different concentrations noting the emergence of peaks around 560 and 672 nm, which belong to PCB_BR_* and the long-living photoproducts X* (see below). Upon loading 0.45% (*w*/*w*) of the dye on plastic, the band assigned to bilirubin-like (PCB_BR_) species predominates the spectral profile. However, PL intensity of the long-living photoproduct increased by ~100-fold (quantum yield of 1.35%, see below), and the excited-state lifetime value extended to ~90 ns (see below) upon loading 6.92% (*w*/*w*) of the dye on plastic. It is not surprising to observe emissions from PCB_BR_*in media of high rigidity (here, we presume the plastic interior environment or glycerol) considering similar findings when PL of PCB was examined in protein-bound forms (phytochrome and cyanobacteriochrome) [10]. However, when more significant amounts of PCB were loaded on plastic the intensity of the Q-band increased with a concomitant decrease in the intensity of the PCB_BR_* emission, which requires further exploration. 

Figure S7 shows a more pronounced and substantial change in the PL spectra of similar concentrations of PCB dyes when adsorbed on top of different electrode membranes, such as AHA and Nafion, compared to the result upon the adsorption of the dye on LDPE plastic. With the addition of dyes to AHA or Nafion under vacuum, new distinct peaks with an entirely different profile have evolved in the blue slide. 

Time-resolved photoluminescence (TRPL) measurements (Table 2) induced via excitation of all PCB species at 510 nm in methanol revealed three-lifetime components with τ_1_ = ~0.1 ns and τ_2_ = ~2 ns associated with PCB_BR_ species (550 and 560 nm, see below) and τ_3_ = ~5 ns pertinent to the long-living photoproduct X* (672 nm). The assignment is based on the previous findings in methanol by other researchers [9,10] that PCB_B_* and PCB_A_* species decay within a few picoseconds, and that the long-living photoproduct X* generated in femtoseconds from PCB_B_* decay in nanoseconds. The picoseconds values for PCB_B_* and PCB_A_* species and the femtoseconds rise time for X* cannot be resolved by our time-resolved PL measurements using our setup that has a time resolution of 30 picoseconds. Triexponential emission decays were also collected for PCB_BR_* (550 and 560 nm) and X* photoproduct (672 nm) upon exciting different samples of PCB while adsorbed on the plastic surface using a diode laser at 510 nm (Experimental Section). 

**Figure 3:**
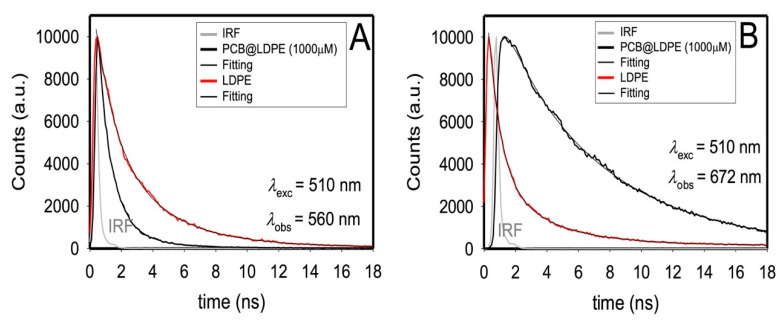
PL decays of solid 1000 µM-PCB@LDPE films at 298 K (black lines). The excitation and monitoring wavelengths are indicated directly in the graph (**A** and **B**). The data for LDPE (red lines) are added for comparison purposes.

Figure 3 and Table 2 show that the average lifetime measured for 1000 µM-PCB-loaded LDPE films depends on the monitored wavelength. In Figure 3, the emission decay for unbound LDPE film was also added for comparison (the data might be associated with scattered photons and not an actual emission). In Table 2, it is also noted that with the increase in the loaded amounts of PCB from 10 µM to 1000 µM on LDPE films, the average lifetime monitored at 560 nm decreased by half (viz. from 2.2 versus 1.3 ns) yet tripled at 672 nm (viz. 1.7 versus 6.7 ns), which requires further exploration. Furthermore, the lifetime and contribution of each component measured for PCB-loaded LDPE films depend on the monitored wavelength (Table 2), which warrants DAS analysis to assign each lifetime to a specific PL maximum (Experimental section). Although emission decays were measured to better comprehend the nature of the fluorescent species in the solid state upon increasing the loaded amount of PCB on plastic (Figures S8–S10 in the Supporting Information and Figure 3), the number of fluorescent species and their emission spectra can only be retrieved through the advanced kinetic analysis. The DAS advanced kinetic analysis unfolded the effects of linking PCB to LDPE plastic on the PCB band, noting that the decay collected at 672 nm (X* photoproduct) is more significantly affected by PCB loading than the one that appeared at 560 nm (PCB_BR_*). 

**Figure 4:**
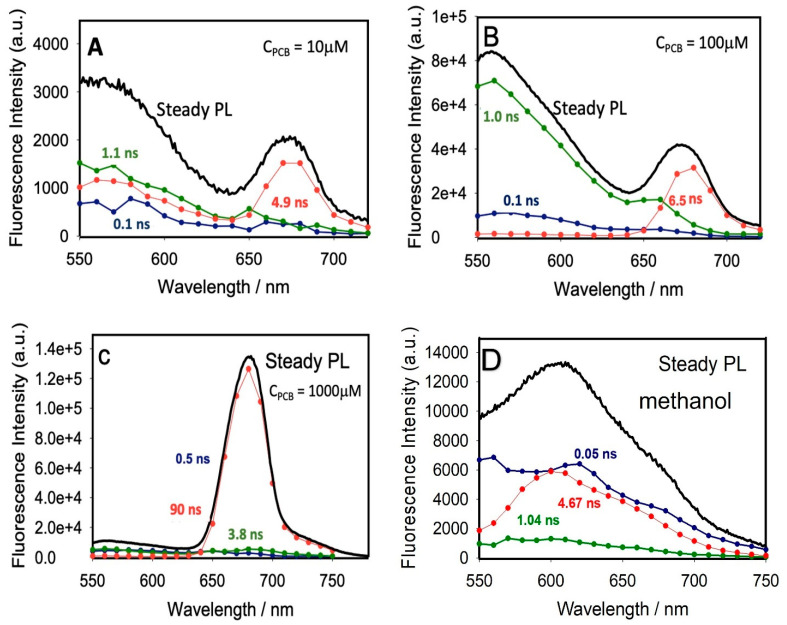
DAS of solid PCB@LDPE films at 298 K (excitation at 510 nm). The total concentrations of PCB and global excited-state lifetimes are indicated directly in the graph (**A**–**D**). DAS spectra in methanol are added for comparison.

In Figure 4, the excited-state lifetime values of PCB for all components and their contributions were retrieved globally at all wavelengths (Experimental section), generating decay-associated spectra (DAS) for all components. For comparison purposes, we observed unresolved DAS spectra for the solution of PCB in methanol (Figure 4D), which highlights the advantage of using LDPE plastic with PCB. Amplitudes and maxima of DAS for all lifetime components of solid PCB@LDPE films are compiled in Table 3. Furthermore, Figure 4 and Table 3 suggest a transfer of the PCB_BR_*’s population (τ_1_ = ~0.1 ns at 550 nm and τ_2_ = ~1 ns at 560) into the long-living photoproduct X* upon increasing the loaded amount of PCB dye (τ_3_ = ~90 ns at 675 nm).

Aimed at developing ionic solar cells based on plastic waste/Nafion composite as the electrode membrane, the work demonstrated the adsorption of PCB photoacids to LDPE film. The transient PL measurements suggest dynamic interactions between PCB and LDPE, which potentially promote the generation of ions (protons). Several reports in the literature examined the structure and dynamic of PCB in organic media, and it was concluded that PCB dye mainly exists in different conformations in the exited state [14,15,16,17,18], leading to its application as a molecular switch [19]. Here, the photophysics of PCB in neat solvents and in the solid state was examined along with the PL and DAS results confirming that the plastic is bound to PCB_BR_* species by analogy to the protein-PCB adduct. At the same time, LDPE plastic promotes the generation of long-living photoproduct X* species when a larger amount of PCB is loaded (from ~0.45 to 7% *w*/*w*) on the surface, as manifested in an increase in the intensity of the Q-band (600–700 nm) at the expense of the intensity of PCB_BR_* species at 400–550 nm. Because the long-living species is formed via the deprotonation of PCB* [10], we postulate that linking plastic waste to natural PCB dye could potentially improve the ionic solar efficiency of the envisaged PCB@LDPE/Nafion electrode. 

On a separate note, and as a control experiment (data not shown), we tested the loading of C-phycocyanin (CP) dye on plastic. We concluded that the pigment could not penetrate the plastic-packed surface while linked to the protein, which has a size mismatch. We have also confirmed that LDPE retains its thermal stability upon loading PCB dyes (Figure S11 in the Supporting Information). Furthermore, we measured the absolute quantum yield for 7%-loaded-PCB LDPE films to be 1.35% (Figure S12 in the Supporting Information). 

For comparison purposes to relevant literature, we noted that in one study PCB was also linked to albumin [20] for enhancing the stability of the latter, and that others utilized other industrial and natural dyes on top of plastic films for other applications such as melamine triazine on LDPE [21] and *Terminalia chebula* on PET [22] for cable and antibacterial applications, respectively. Another work applied one natural dye extracted from Azadirachta indica (neem) in solar cell technology [23]. We envisage the advantages of our research are that it deploys natural photoacids for the more recently emerged ionic solar cell technology. 

## 3. Conclusions

Utilizing optical measurements in the liquid and solid state, we confirmed in the present work that dyeing polyethylene (PE) plastic waste with a bioactive photoacid phycocyanobilin (PCB) extracted from the pigment of Spirulina blue–green algae improves the populations of long-living photoproducts X*, which are pertinent to deprotonated pyrrole species (anionic species). The selected LDPE plastics have low recycling but high production rates. Here, the results demonstrated the potential benefits of these solid wastes for constructing ionic solar cells when dyed with natural photoacid dyes in the future.

## 4. Method 

### 4.1. Chemical and Reagent 

C-phycocyanin dye (CP), PCB dye, organic solvents, and LDPE plastic film were purchased from Sigma-Aldrich, Saint-Louis, MO, USA. The Nafion 117 film (Chemours) was procured from Fuel Cell Store, TX, USA and AHA (Neosepta) was obtained from ASTOM, Tokyo, Japan. 

### 4.2. Synthesis of PCB-Bound LDPE Films

Specific sizes of LDPE films (Table S5, Supporting Information) were immersed in different concentrations of PCB and CP dyes in methanol under a vacuum for one week. As a control experiment, different solvent mixtures of water and methanol were also tested: PCB in MeOH of 10, 100, and 1000 µM concentrations; PCB in methanol–water (50%) mixture of 1000 µM concentration; CP in water of 1000 µM concentration; CP in water–methanol (50%) mixture of 1000 µM; and CP in methanol of 10, 100, and 1000 µM concentrations. The final film was washed using the same solvents. The same experiment was repeated for Nafion and AHA membranes (PCB in MeOH of 1000 µM). 

### 4.3. DSC Measurement

The thermal response of each sample was measured using a differential scanning calorimeter (Shimadzu DSC-60 Plus, Kyoto, Japan). About 5–6 mg of sample was precisely weighed into DSC sample pans which were sealed by means of a pellet press with a top lid. Samples were measured in the temperature range of 30–350 °C at a heating rate of 10 °C/min under a constant nitrogen flow of 50mL/min. Data analysis was performed using the *Lab solutions TA* software, USA.

### 4.4. XPS Measurement

The PCB-sensitized polymer film was subjected to Al Kα X-ray radiation (1486.6 eV) under ultra-high vacuum conditions using a photoelectron spectrometer (model JPS-9030, JEOL, Tokyo, Japan). The X-ray beam was normal to the film. This resulted in photoelectrons, which were collected at an angle of 45° to the direction of the X-ray beam. For the survey spectrum, the step was 1 eV with a scan of 2 for the Nafion sample and 5 for the LDPE sample; the step was 0.1 eV with a scan of 5 or 10 for C1s, O1s, and F1s elements, and 60 for S2p element. The spectrum was subjected to a relative referencing correction for the effect of charging, leading to a shift in the spectrum to the higher binding energies. The peak was fitted using the Gauss function of the ORIGIN program (USA) for determining the peak position (Supplementary Information). 

### 4.5. Excited-State PL lifetime Measurements

The PL and PLE spectra for PCB in neat solvents and for the solid films were measured on FS5 (Edinburgh Instrument) with *λ*_ex_ of 400 nm and *λ*_em_ of 672 nm, featuring Hamamatsu H5773-04 detector (R928P detector; Edinburgh Photonics, Edinburgh, UK). Emission decays were also measured at 672 nm using time-correlated single-photon counting (TCSPC)-based Edinburgh Instrument (LifeSpec II spectrometer, Edinburgh, UK). A picosecond diode laser with *λ*_ex_ of 510 nm and a long pass filter (LPF) at 530 nm was employed to collect PL decays with a total count rate of 10,000 counts/s by using a red-sensitive high-speed PMT detector (H5773-04, Hamamatsu, Japan). Utilizing least-square statistical analysis (chi-square and residual plot to assess the goodness of fit), the average lifetime ($\bar{\tau }$) was then calculated using ($\bar{\tau }=\sum_{i}f_{i}\tau_{i}$) where f_i_ is the extracted intensity–contribution fraction. The experimental error was estimated to be around 2–5% depending on the lifetime values. 

### 4.6. Absolute PL Quantum Yield (QY) Measurements

Absolute QY was estimated for the solid films on the FS5 spectrometer by utilizing an integrating sphere (SC-30) and by comparing the measured direct and indirect emission from the sample to that generated from the PTFE reference through direct excitation. The bandwidths were kept at 3 and 0.5 for the excitation and emission monochromators. The sample was excited at 400 nm. 

### 4.7. DAS Measurements

The emission decays collected every 10 nm over the entire emission spectra of PCB-bound LDPE films with a dwell time of 10 s at each wavelength were globally fitted with a tri-exponential model function, then convoluted with an instrument response function (IRF) of ~30 ps. The time-resolved data were specifically analyzed using Edinburgh FAST software (UK), in which decay-associated spectra (DAS) were constructed from the extracted intensity-contribution fraction (*f_i_*) calculated from the pre-exponential amplitudes (*B_i_*), as follows:

(1) $$I\left(t\right)=\underset{i}{\sum }B_{i}exp\left(-t/\tau_{i}\right)$$

(2) $$f_{i}=\frac{B_{i}\tau_{i}}{\sum_{j}B_{j}\tau_{j}}$$

### 4.8. Loading Characterization Methods

The loading of PCB guest on LDPE film was estimated using UV−vis absorption spectroscopy and calibration curves obtained from known concentrations of PCB dye in methanol (Supporting Information). 

## 5. Supporting Information

The Supporting Information is available free of charge at PL, PLE, and TRPL spectra, XPS spectra of PCB-sensitized Nafion; XPS spectra of PCB-sensitized LDPE; Calibration curves and loading measurements; Changes of PL spectra of PCB-sensitized electrolytic membranes; PL decays of solid PCB@LDPE films; DSC curve of solid PCB@LDPE films; Absolute quantum yield measurements, PL, and PLE spectra of solid PCB@LDPE films; References.

## Figures and Tables

**Table 1:** PLE (λ_e_) and PL (λ_f_) peak positions, average excited-state lifetimes (τ), and stokes shifts (Δν) for PCB dye at 10 µM in several neat solvents at 298 K. Solvent viscosity (η) is also included.

Solvents	Soret-Band *λ*_e_ (nm) ^a^	Q-Band *λ*_e_ (nm) ^a^	PCB_BR_ *λ*_f_ (nm) ^b^	Q-Band *λ*_f_ (nm) ^b^	Δν (cm^−1^)	*τ* (ns) ^c^	η (cP)
Diethyl ether	406	563	468	671	2859	1.82	0.24
Acetonitrile	406	560	502	670	2932	2.18	0.34
Acetone	406	561	501	671	2922	1.98	0.36
Dichloromethane	412	563	490	674	2925	2.12	0.41
Ethyl acetate	406	560	486	672	2977	1.74	0.45
Methanol	404	562	485	671	2890	2.44	0.54
Dioxane	409	560	470	674	3021	2.30	1.37
Dimethyl sulfoxide	410	560	485	674	3221	2.48	1.99
Butanol	402	562	530	673	2935	1.25	2.57
Chlorobenzene	416	565	470	675	1580	1.63	7.68
Mineral oil	411	563	465	674	1503	1.30	84
Glycerol	350	-	462	-	6926	1.04	954

^a^ The dye was excited at 400 nm in all solvents for the PL measurements. ^b^ PLE spectra were collected for emission peak at 670 nm. ^c^ For the TRPL measurements, excitation was carried out at 510 nm (with a time resolution of 30 picoseconds), and the emission was monitored at 670 nm. The average excited-state lifetime was calculated as described in the Experimental Method.

**Table 2:** The observed excited-state lifetime of PCB-loaded LDPE films (PCB in methanol were added for comparison).

Samples	λ_obs_ (nm)	τ_1_ (ns)	f_1_%	τ_2_ (ns)	f_2_%	τ_3_ (ns)	f_3_%	τ_average_ (ns)	*Chi-Square*
PCB in MeOH (10 µM)	560	0.1	40	1.9	22	5.2	38	2.4	1.034
	672	0.1	42	1.8	13	5.2	45	2.6	0.985
PCB@LDPE (10 µM)	560	0.6	55	2.8	35	8.7	10	2.2	1.404
	672	0.3	64	1.5	16	6.1	20	1.7	1.148
PCB@LDPE (100 µM)	560	0.8	75	1.7	24	15.6	1	1.2	1.090
	672	1.1	26	6.6	71	16.6	3	5.5	1.240
PCB@LDPE (1000 µM)	560	0.4	30	1.2	60	4.7	10	1.3	1.333
	672	1.1	2	5.2	38	7.8	60	6.7	1.168

The time resolution was ~30 picoseconds, and the excitation wavelength was 510 nm.

**Table 3:** Excited-state lifetime values, DAS maxima, and amplitudes extracted from DAS global analysis (excitation at 510 nm).

% Loaded PCB/per LDPE (*w*/*w*)	*τ*/ns Excited-State Lifetimes			DAS Maximum (nm)			DAS Amplitudes			%X*
	PCB_BR_* 1	PCB_BR_* 2	X*	PCB_BR_* 1	PCB_BR_* 2	X*	PCB_BR_* 1	PCB_BR_* 2	X*	
0.08%	0.1	1.1	4.9	550	560	675	0.20	0.50	0.30	30%
0.44%	0.1	1.0	6.6	550	560	675	0.05	0.65	0.30	30%
6.92%	0.5	3.8	90	550	560	675	0.05	0.05	0.90	90%

## Supplementary Materials

The following supporting information can be downloaded at: https://www.mdpi.com/article/10.3390/polym14224811/s1, Figure S1: PLE (blue) and PL (red) spectra (λ_exc_ = 400 nm and λ_mon_ = 672 nm) of PCB dye in different solvents at 298 K. The name of the solvent is directly indicated in the paragraph; Figure S2: PL decays (red) at 672 nm and the three-exponentials nonlinear fitting (red) spectra (λ_exc_ = 510 nm with 30 picoseconds time-resolution) of PCB dye in different solvents at 298 K. The name of the solvent and the average lifetime values are directly indicated in the paragraph. The instrument response function (IRF; gray) is also shown in the paragraph; Table S1: Solvent properties: refractive index (n), dielectric constant (ε), orientational polarizability (Δf), viscosity (η), hydrogen bond accepting ability (β), hydrogen bond donating ability (α), and polarity/polarizability parameter (π*); Figure S3: XPS spectra for (a) survey and (b) elements (C1s, O1s, F1s, and S2p) of PCB-sensitized Nafion. The inset shows the chemical structure of PCB; Figure S4: Best fit for elements (C1s, O1s, F1s, and S2p) of PCB-sensitized Nafion; Table S2: Binding energy in eV for elements (C1s, O1s, F1s, and S2p) of PCB-sensitized Nafion; Figure S5: Best fit for elements (C1s and O1s) of PCB-sensitized LDPE; Table S3: Binding energy in eV for elements (C1s and O1s) of PCB-sensitized LDPE; Figure S6: The standard equation and the calibration curves obtained from UV−visible absorption spectra were measured for different concentrations of PCB dye in methanol at 298 K; Table S4: Mass-of-balance Analysis; Table S5: Properties of Plastic Film; Figure S7: Changes of PL spectra of LDPE, Nafion, and AHA solid membranes upon the addition of different concentrations of PCB dyes in methanol under vacuum at 298 K (solid samples were excited at 510 nm); Figure S8: PL decays of PCB (10 µM) in methanol at 298 K. The excitation and monitoring wavelength is indicated directly in the graph; Figure S9: PL decays of solid PCB@LDPE films prepared upon loading PCB dyes at 10 µM at 298 K (black lines). The excitation and monitoring wavelengths are indicated directly in the graph. The data for LDPE (red lines) are added for comparison purposes; Figure S10: PL decays of solid PCB@LDPE films prepared upon loading PCB dyes at 100 µM at 298 K (black lines). The excitation and monitoring wavelengths are indicated directly in the graph. The data for LDPE (red lines) are added for comparison purposes; Figure S11: DSC curves of solid PCB@LDPE films prepared upon loading PCB dyes at 100 (red line) and 1000 µM at 298 K (green line). The DSC curve for LDPE (blue line) was added for comparison purposes; Figure S12: (A) PLE (blue) and PL (red) spectra (λ_exc_ = 400 nm and λ_mon_ = 672 nm) of solid PCB@LDPE films prepared upon loading 7% of PCB dyes at 298 K. (B) the measured Quantum Yield (QY) at 672 nm as directly indicated in the paragraph.

## Abbreviations

Neosepta AHA, brand name for an anion exchange membrane; Nafion, a sulfonated tetrafluoroethylene based fluoropolymer-copolymer; Soret-band, an intense peak in the blue wavelength region of the visible spectrum; PCB, Phycocyanobilin; CP, C-Phycocyanin dye; LDPE, low-density polyethylene; PL, photoluminescence spectra; PLE, photoluminescence excitation spectra; TRPL, time-resolved photoluminescence spectra; DAS, decay-associated spectra; XPS, X-ray photoelectron spectra; DSC, differential scanning calorimeter.
