# Supplementary material for: Dyeing Non-Recyclable Polyethylene Plastic with Photoacid Phycocyanobilin from Spirulina Algae: Ultrafast Photoluminescence Studies
Source: Polymers (Basel). 2022 Nov 9;14(22):4811. doi: 10.3390/polym14224811 (PMC9693856; doi:10.3390/polym14224811)
Supplement: Supplementary file 1 [file polymers-14-04811-s001.zip › polymers-1962289-supplementary.pdf]

SUPPORTING INFORMATION

*Maryam Alhefeiti,[1] Falguni Chandra,[1] Ravindra Kumar Gupta,[2] Na'il Saleh.[1]**

[1] *Department of Chemistry, College of Science, United Arab Emirates University, Al Ain P.O. Box 15551, United Arab Emirates*

[2] *King Abdullah Institute for Nanotechnology, King Saud University, Riyadh* 11451, *Saudi Arabia*

[§]*Equal contribution*

TABLE OF CONTENTS

Part 1: PL, PLE, and TRPL Spectra……………………………………………………….S3

PL and PLE Spectra of PCB in neat solvents …………………..…………..…….S3

TRPL Spectra of PCB in neat solvents ……………………………….………..……S5

Table S1: Solvent Properties……………………………………………………..S7

Part 2: XPS Spectra ………………………………………………..…...………..… S8

XPS Spectra of PCB-Sensitized Nafion ……………………..…………..…….S8

XPS Spectra of PCB-Sensitized LDPE……………………………….………..……S11

Part 3: Loading Characterization…………………………………………………..… S13

Calibration Curves and Loading Measurements …………..………….....… S12-13

Part 4: PL and Time-Resolved PL Spectra……………………………….…. S14

Changes of PL spectra of PCB-Sensitized Electrolytic Membranes ……….….... S14

PL decays of solid PCB@LDPE films ……………...……………..……..... S15-17

Part 5: Thermal Stability ……………………………………………………..… S18

DSC curve of solid PCB@LDPE films ……………………..……......... S18

Part 6: Absolute Quantum Yield Measurements…………………………………….S19

PL and PLE Spectra of solid PCB@LDPE films ……………………………19

References ………………………………………………………………………….S20

Part 1: PL and PLE Spectra

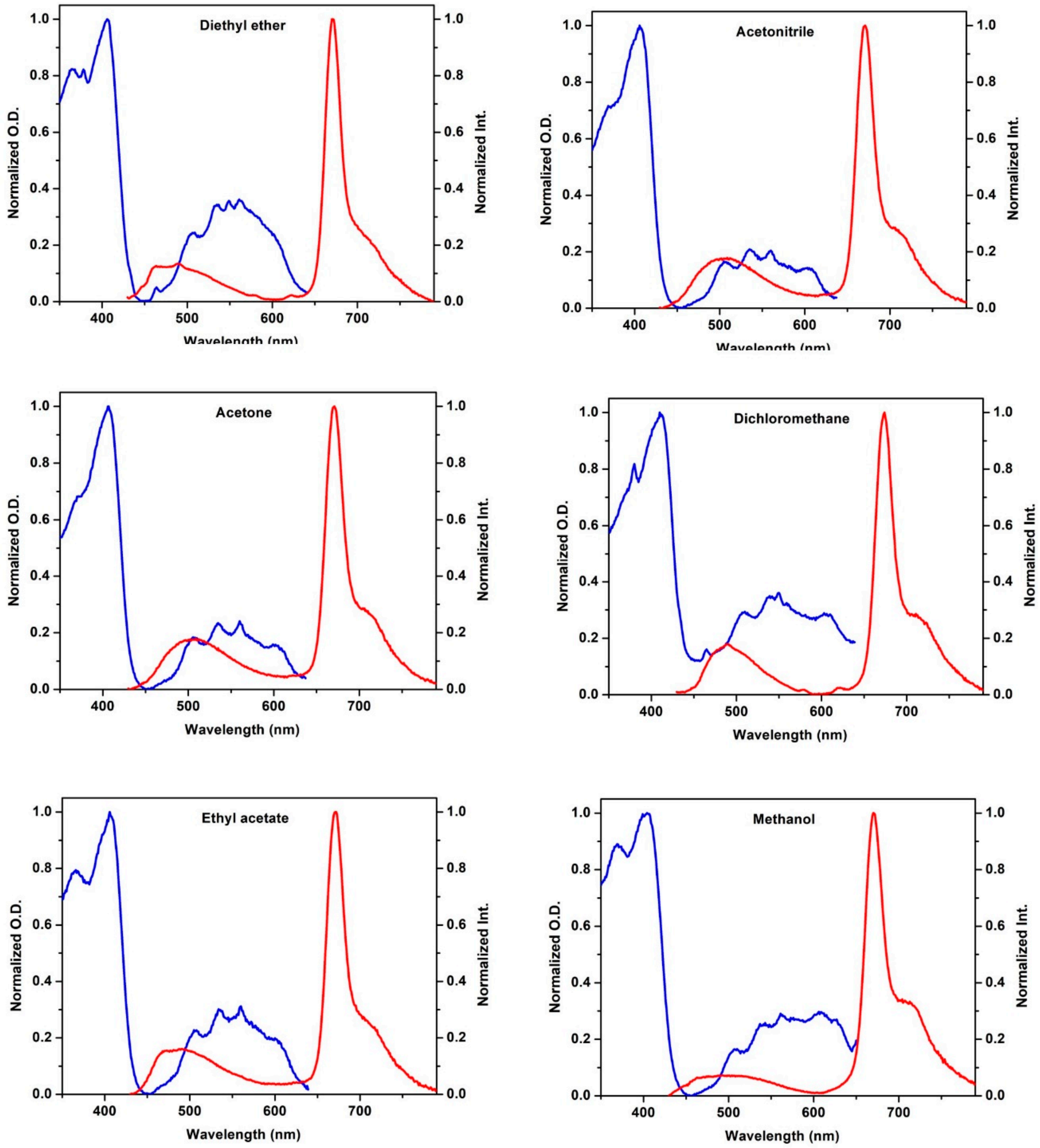

Figure S1. PLE (blue) and PL (red) spectra (λ_{exc} = 400 nm and λ_{mon} = 672 nm) of PCB dye in different solvents at 298 K. The name of the solvent is directly indicated in the paragraph.

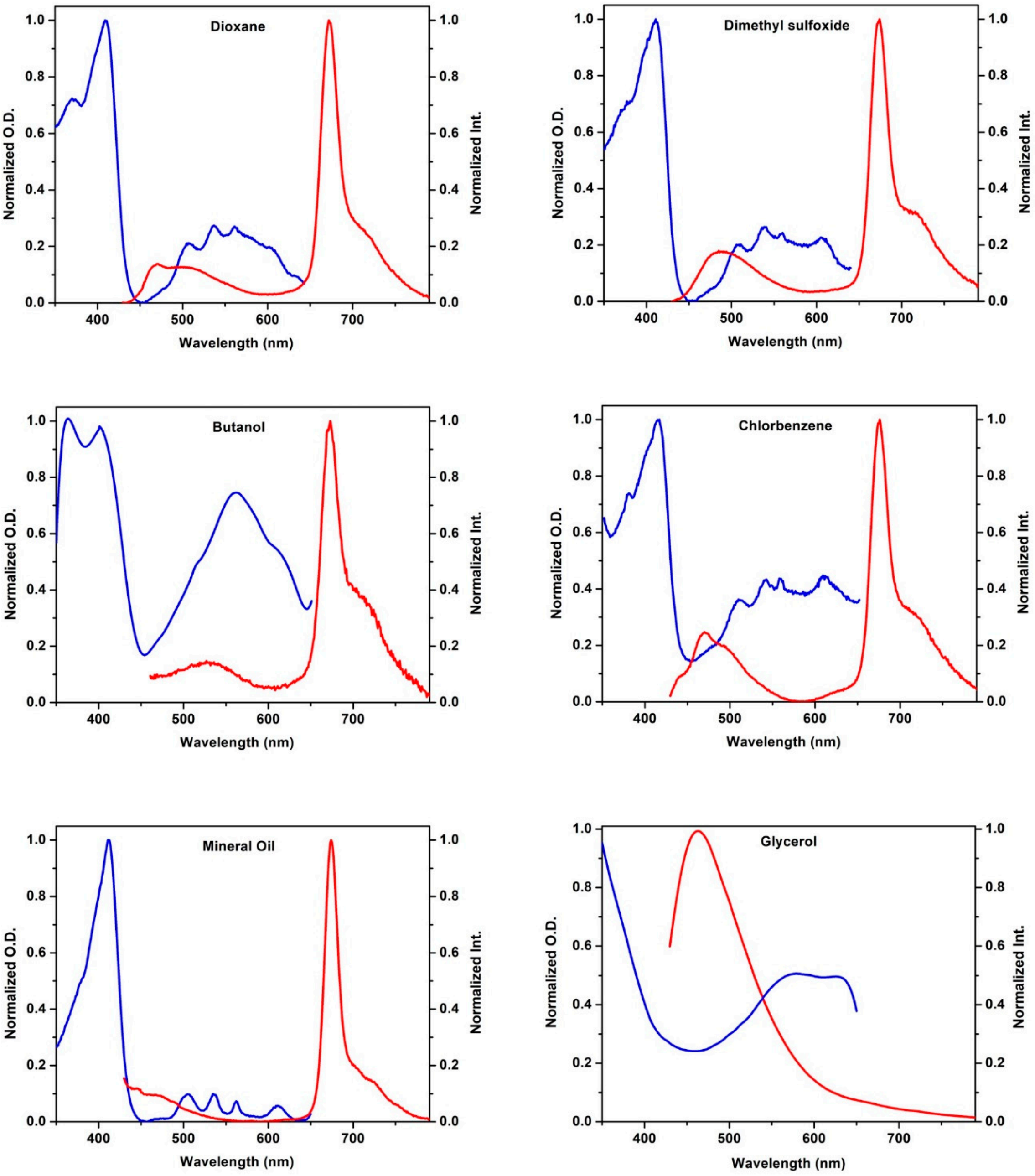

Figure S1. Continued.

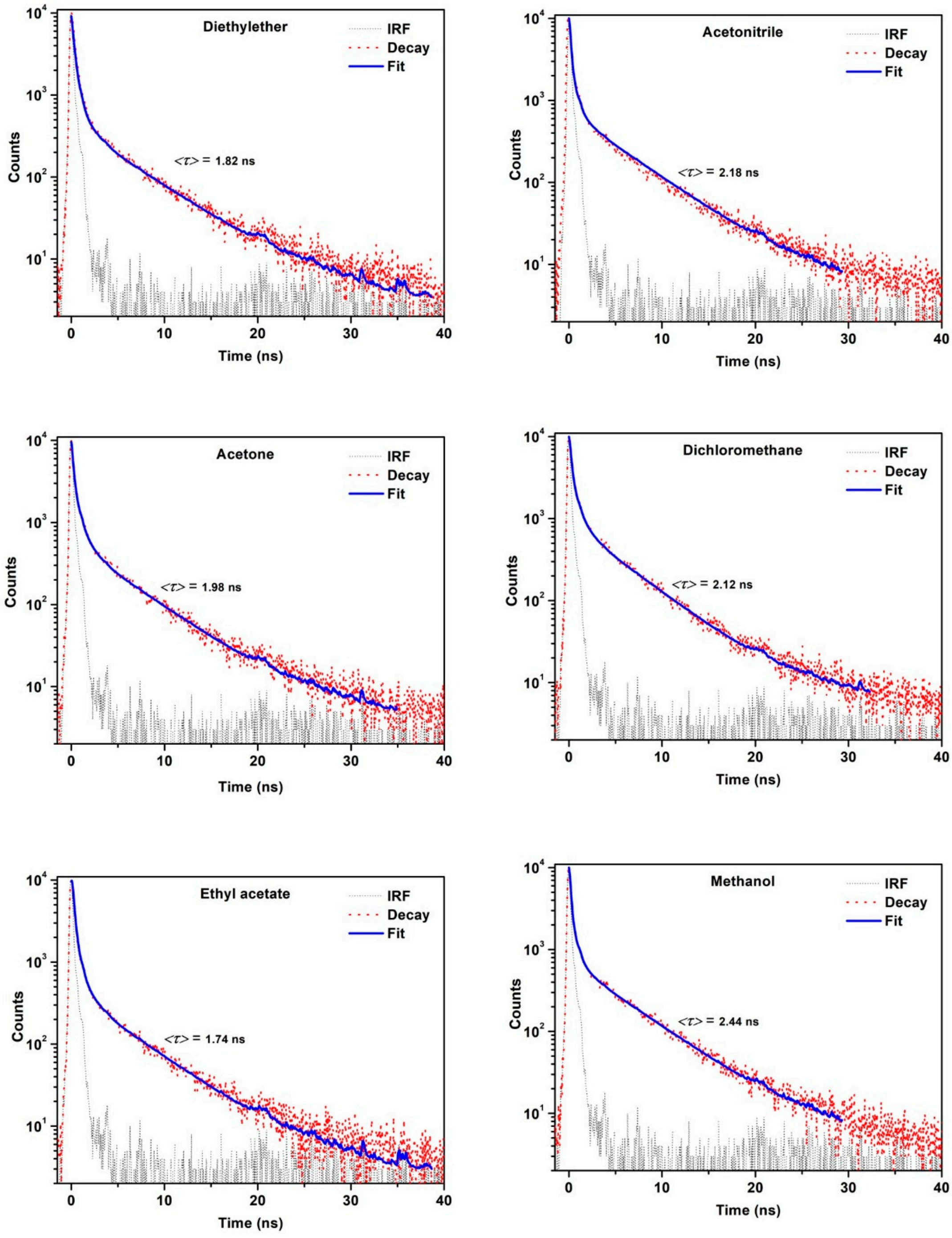

Figure S2. PL decays (red) at 672 nm and the three-exponentials nonlinear fitting (red) spectra (λ_{exc} = 510 nm with 30 picoseconds time-resolution) of PCB dye in different solvents at 298 K. The name of the solvent and the average lifetime values are directly indicated in the paragraph. The instrument response function (IRF; gray) is also shown in the paragraph.

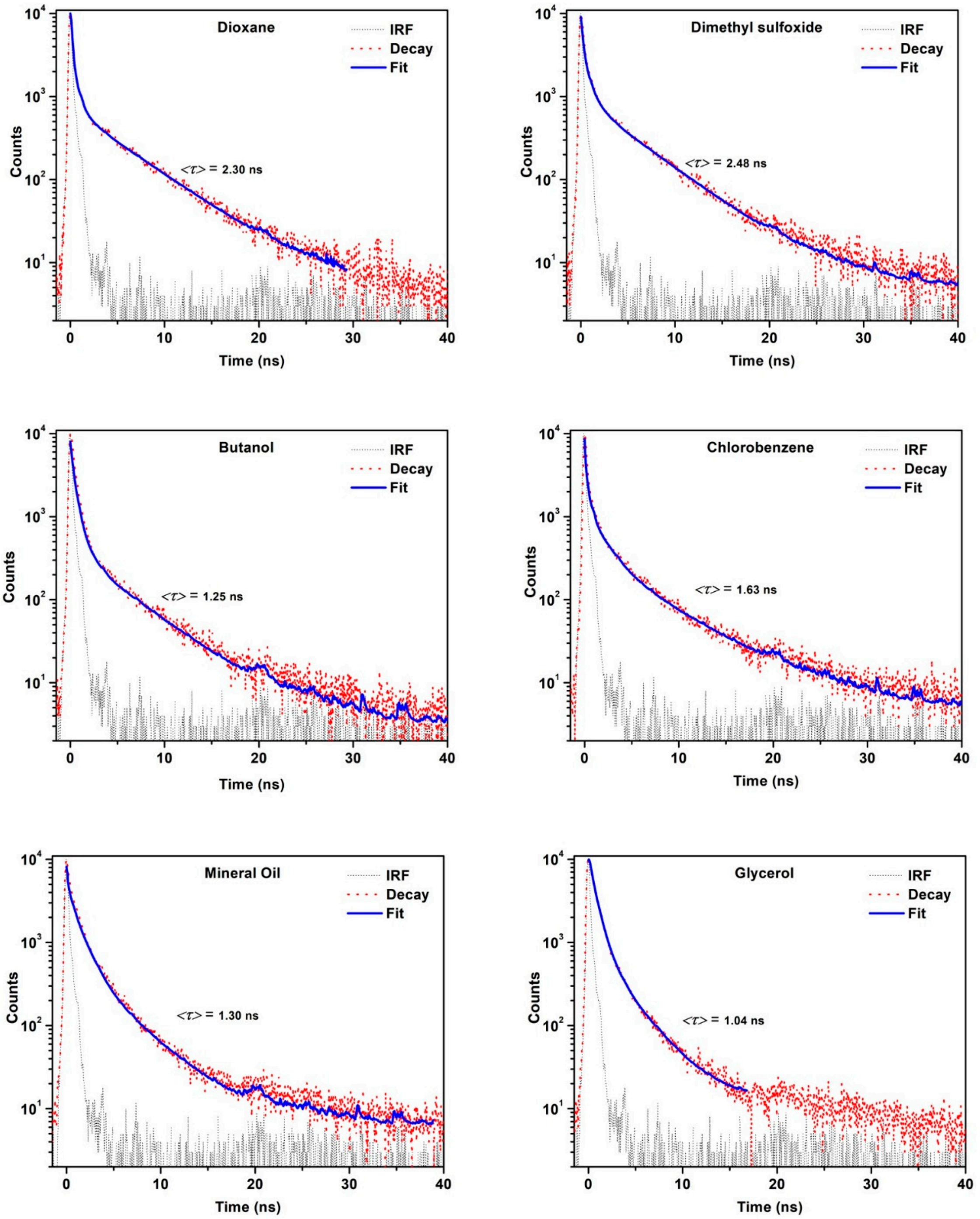

Figure S2. Continued.

Table S1. Solvent properties: refractive index (n), dielectric constant (ε), orientational polarizability (Δf), viscosity (η), hydrogen bond accepting ability (β), hydrogen bond donating ability (α), and polarity/polarizability parameter (π*).

Solvents	n	ε	Δf	α	β	π*	α + β + π*
Diethyl ether	1.352	4.33	0.17	0	0.47	0.27	0.74
Acetonitrile	1.344	37.5	0.31	0.19	0.31	0.75	1.25
Acetone	1.35	20.70	0.29	0.08	0.48	0.71	1.27
Dichloromethane	1.416	8.93	0.22	0.33	0	0.82	1.15
Ethyl Acetate	1.37	6.02	0.17	0	0.45	0.45	0.90
Methanol	1.328	32.7	0.31	0.93	0.62	0.6	2.15
Dioxane	1.422	2.22	0.02	0	0.37	0.55	0.92
Dimethyl sulphoxide	1.479	48.9	0.26	0	0.76	1	1.76
Butanol	1.394	17.85	0.27	0.79	0.88	0.47	2.14
Chlorobenzene	1.52	5.62	0.14	0	0.44	0.19	0.63
Mineral Oil	1.47	2.22	0.01	NA	NA	NA	NA
Glycerol	1.46	46.50	0.27	0.93	0.67	0.04	1.64

NA: not applicable.

Part 2: XPS and PL Spectra

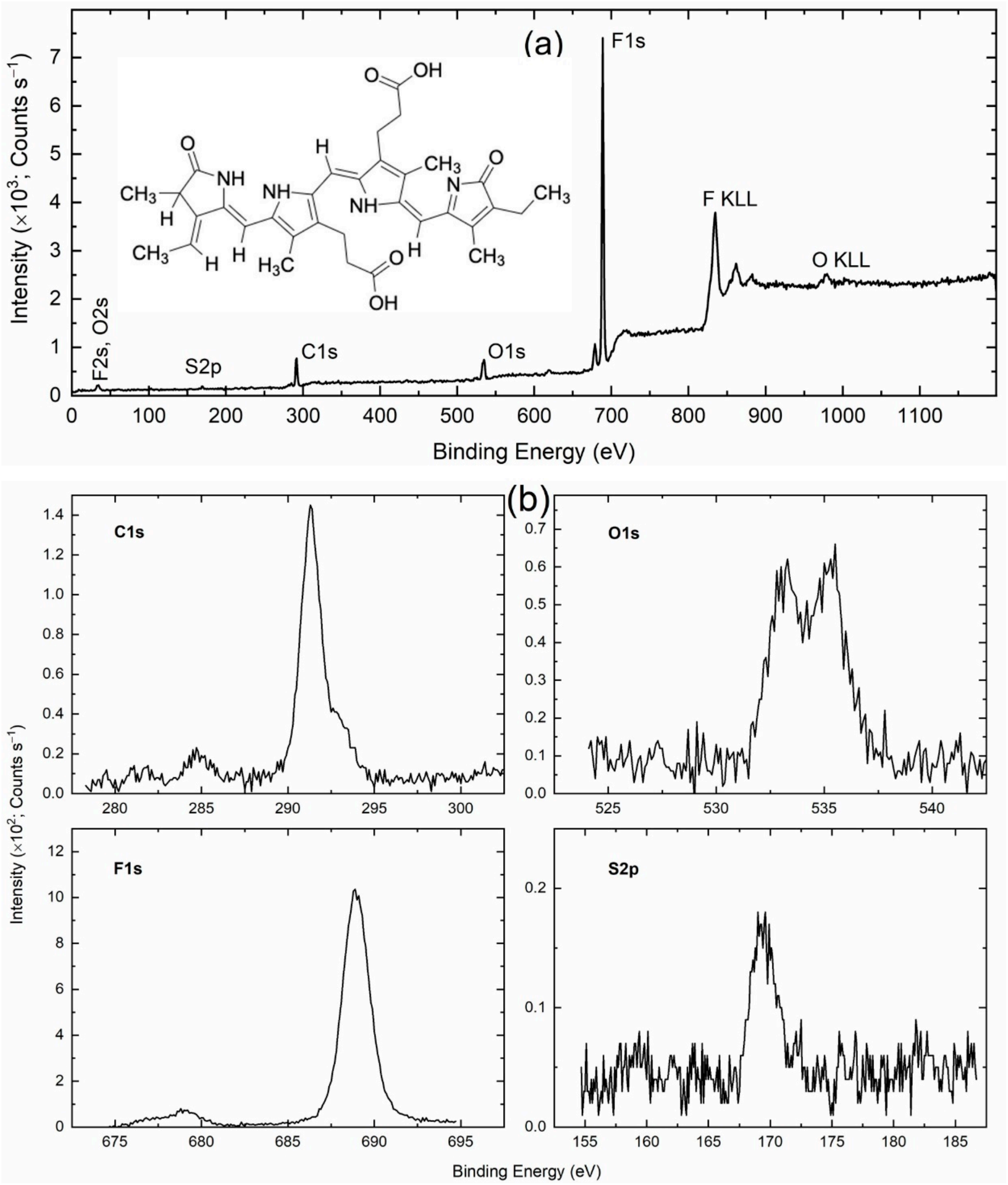

Figure S3. XPS spectra for (a) survey and (b) elements (C1s, O1s, F1s, and S2p) of PCB-sensitized Nafion. The *inset* shows the chemical structure of PCB.

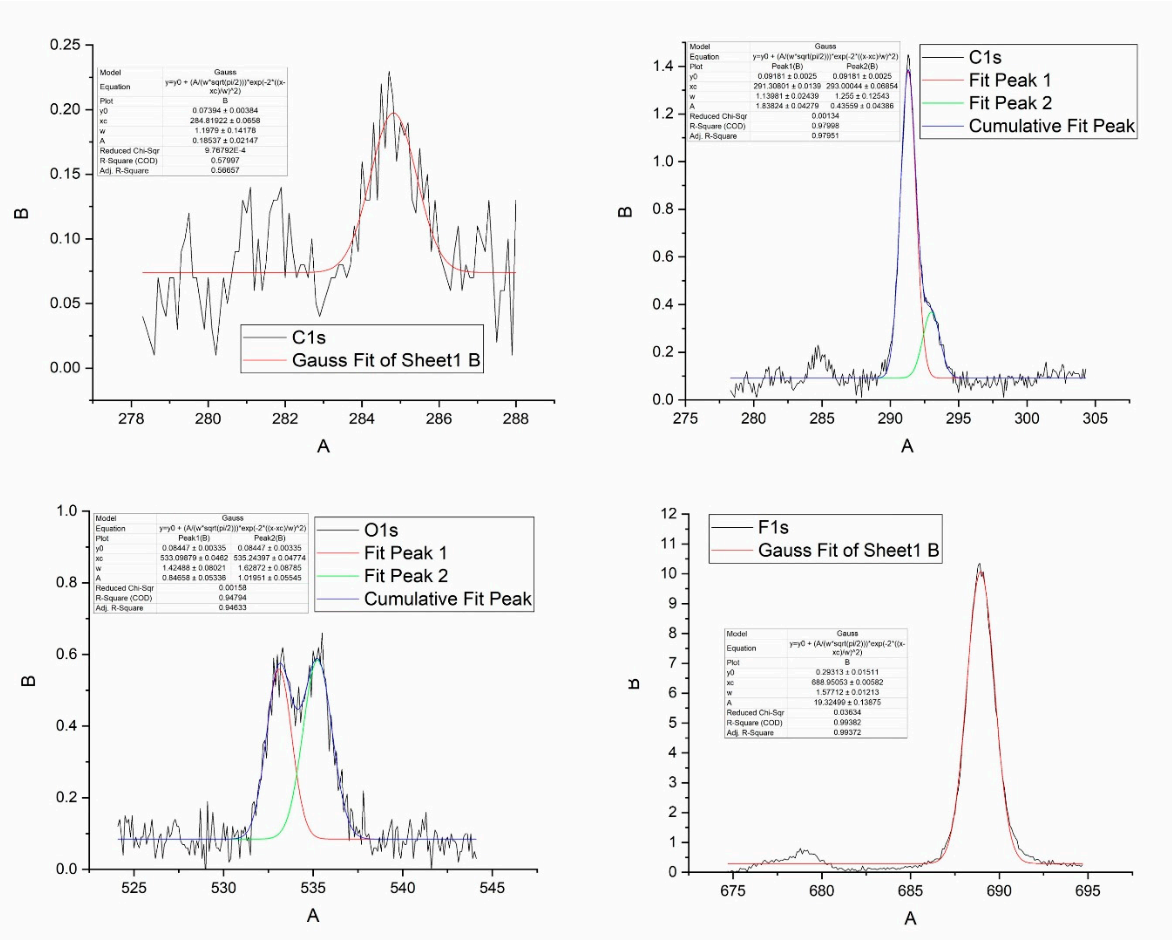

Figure S4. Best fit for elements (C1s, O1s, F1s, and S2p) of PCB-sensitized Nafion.

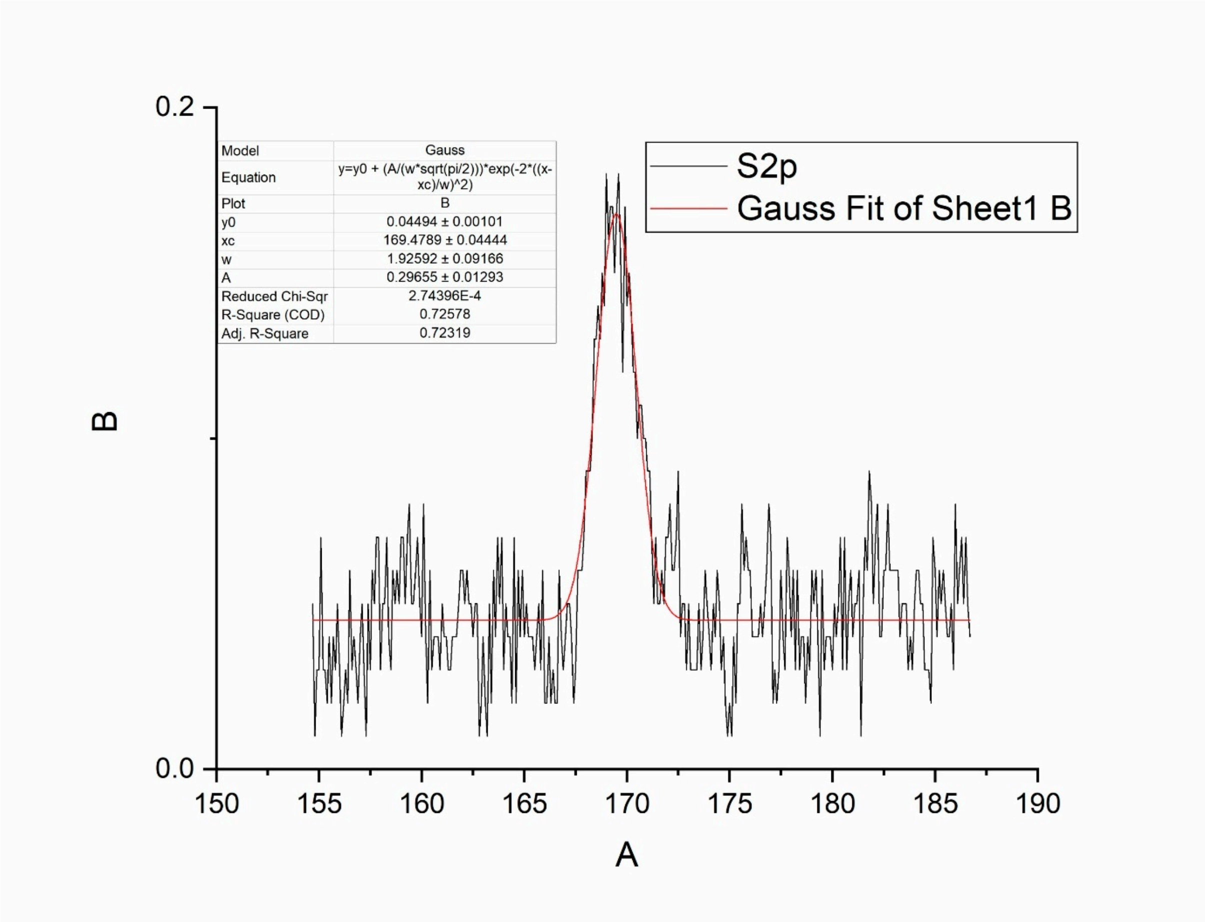

Figure S4. Continued.

Table S2. Binding energy in eV for elements (C1s, O1s, F1s, and S2p) of PCB-sensitized Nafion.

C1s	284.8 (284.8, C–C)†;<br>291.3 (292.2, –CF2–);<br>293 (294.4, –CF3)
O1s	533.1 (533, $–SO_3^-$)†,§;<br>535.2 (535.7, –CF2–O–CF–)
F1s	688.9 (689, C–F)†
S2p	169.5 (170, $–SO_3^-$)†

† [1,2], § [3]

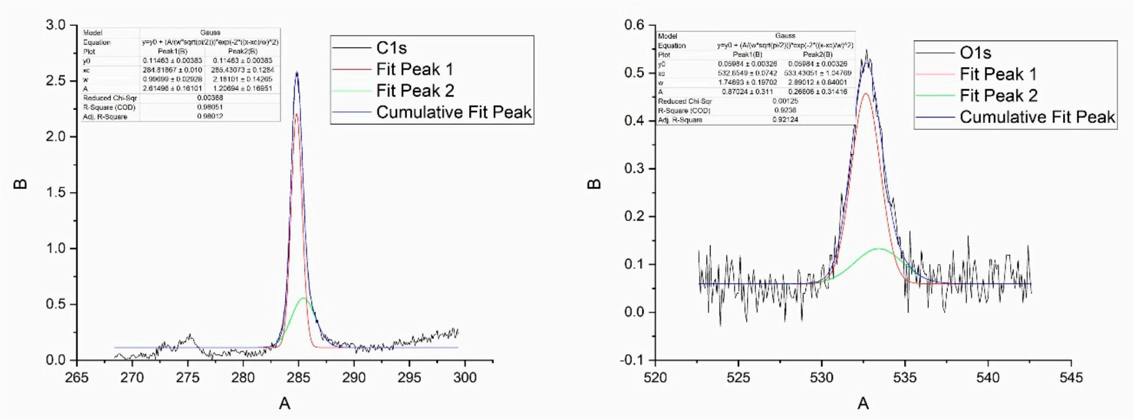

Figure S5. Best fit for elements (C1s and O1s) of PCB-sensitized LDPE.

Table S3. Binding energy in eV for elements (C1s and O1s) of PCB-sensitized LDPE.

C1s	284.8 (284.6, C–C) ‡;<br>285.4 (286, C–O) §
O1s	532.6; 533.4 (C=O; COOH)§

‡ [4], § [3]

Part 3: Loading Characterization

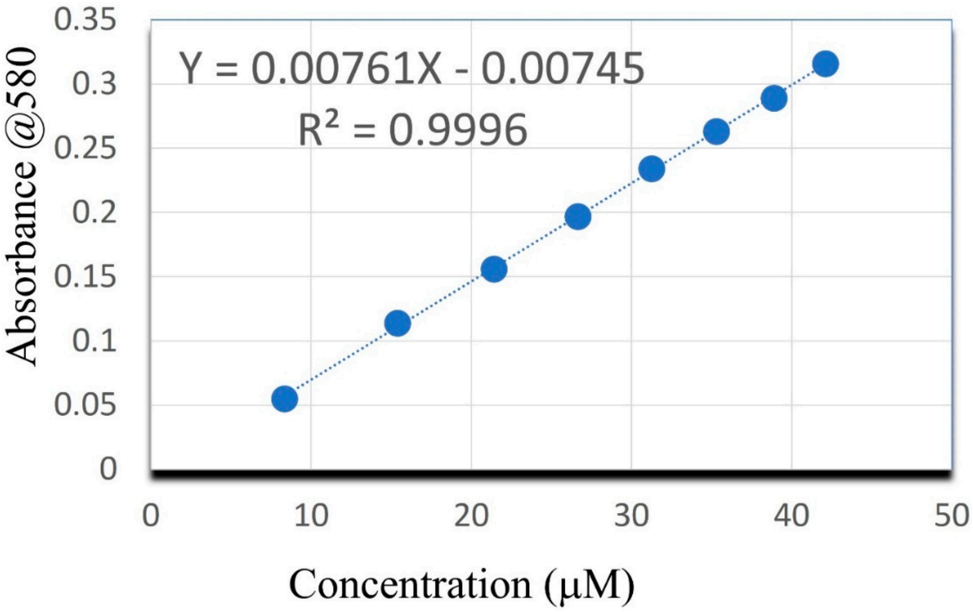

Figure S6. The standard equation and the calibration curves obtained from UV−visible absorption spectra were measured for different concentrations of PCB dye in methanol at 298 K.

Table S4. Mass-of-balance Analysis.

Y (Absorbance)	X =[PCB] (μM)	C_{PCB} (μM)	[PCB@LDPE] (μM)	**Weight (g) of PCB** ± 0.001[§]
~0	~0	< 10	< 10 (in 0.002 L)	< 0.000012 (in 0.002 L)
0.360545	48.36	100	51.64 (in 0.002 L)	0.000060 (in 0.002 L)
0.405661	54.28	1000	945.72 (in 0.0017 L)	0.000942 (in 0.0017 L)

[§]Total amount used of PCB = 0.001000 g (molecular weight = 585.714 g/mol)

The concentrations (number of moles) of PCB dye in the remaining and washed-out solutions ([PCB]) after the loading step (Experimental Section) were calculated by replacing Y (Figure S6) in the standard equation S1 by the absorbance at 580 nm for a given solution (Table S4).

$$X = \frac{Y+0.00745}{0.00761} \quad \text{(Equation S1)}$$

The following mass-of-balance (equation S2) was then used to calculate the amount of PCB on top of LDPE film (Table S4: **Weight (g) of PCB**).

$$C_{PCB} = [PCB] + [PCB@LDPE] \qquad \text{(Equation S2)}$$

, where C_{PCB} is the total concentration of PCB, [PCB] is the concentration of the unbound PCB, and [PCB@LDPE] is the concentration of the adsorbed dye onto the plastic surface.

Table S5. Properties of Plastic Films.

**Plastic film**	**Thickness (mm) ± 0.001**	**C_{PCB} (μM)**	**Area (mm^2) ± 1**	**Weight (g) ± 0.001**	**Color**	**Dye Loading (%) (w/w)**
LDPE	0.120	0	100	0.0293	Watery	
PCB@LDPE	0.117	100	100	0.0136	Watery	< 0.08
PCB@LDPE	0.118	100	100	0.0137	Watery	0.44
PCB@LDPE	0.115	1000	100	0.0136	Watery	6.92
PCB@Nafion	0.183	1000	100	0.0240	Dark brown	
PCB@AHA	0.180	1000	100	0.0478	Dark brown	
Nafion	0.170	0	100	0.0366	Watery	
AHA	0.174	0	100	0.0172	Creamy	

Part 4: PL and Time-Resolved PL Spectra

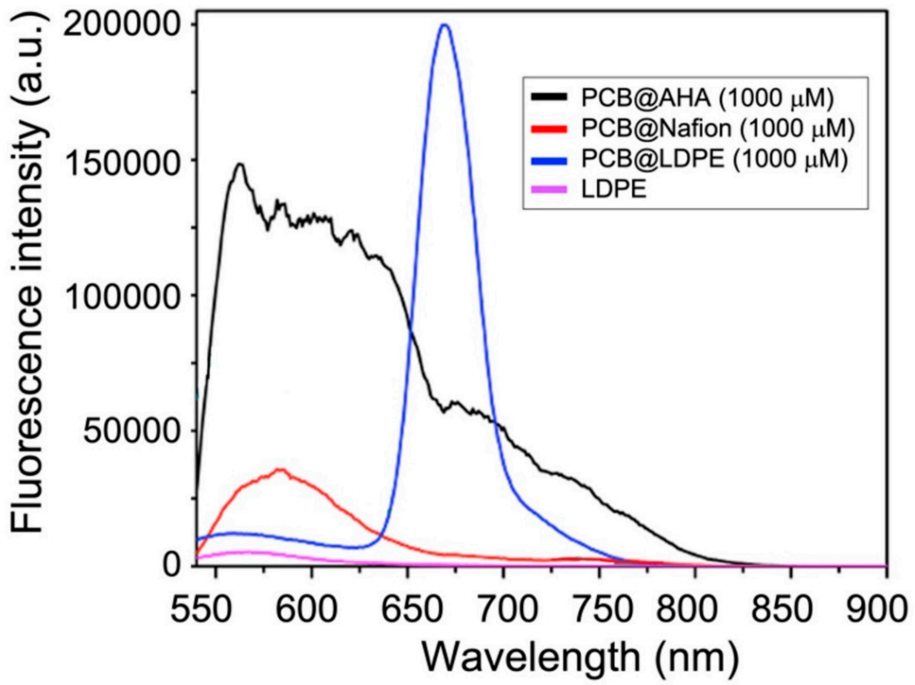

Figure S7. Changes of PL spectra of LDPE, Nafion, and AHA solid membranes upon the addition of different concentrations of PCB dyes in methanol under vacuum at 298 K (solid samples were excited at 510 nm)

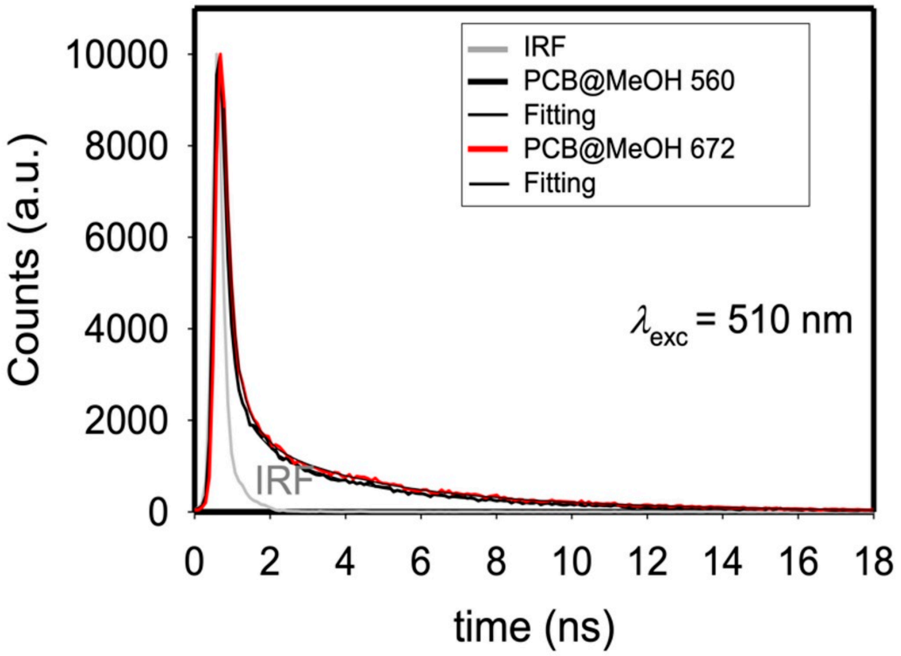

Figure S8. PL decays of PCB (10 μM) in methanol at 298 K. The excitation and monitoring wavelength is indicated directly in the graph.

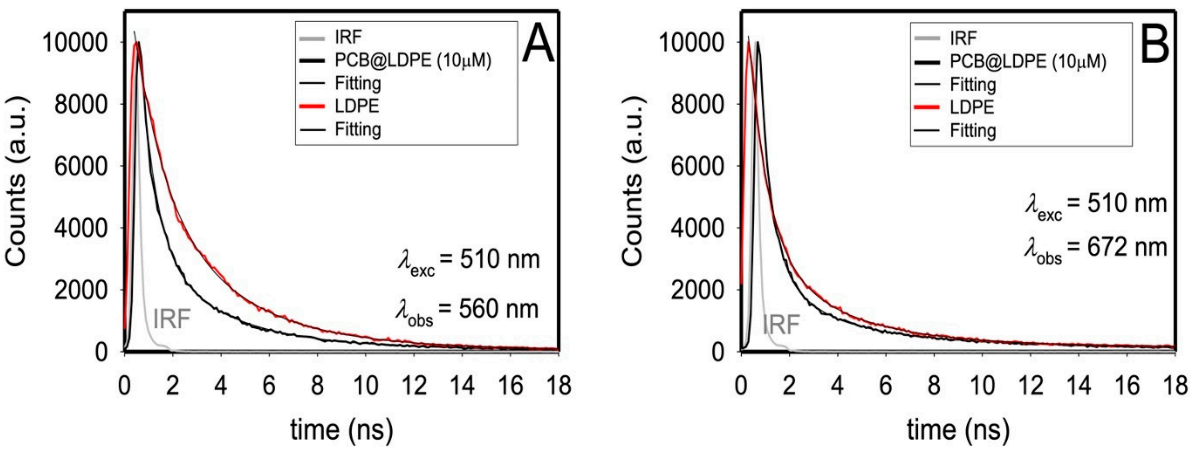

Figure S9. PL decays of solid PCB@LDPE films prepared upon loading PCB dyes at 10 μM at 298 K (black lines). The excitation and monitoring wavelengths are indicated directly in the graph. The data for LDPE (red lines) are added for comparison purposes.

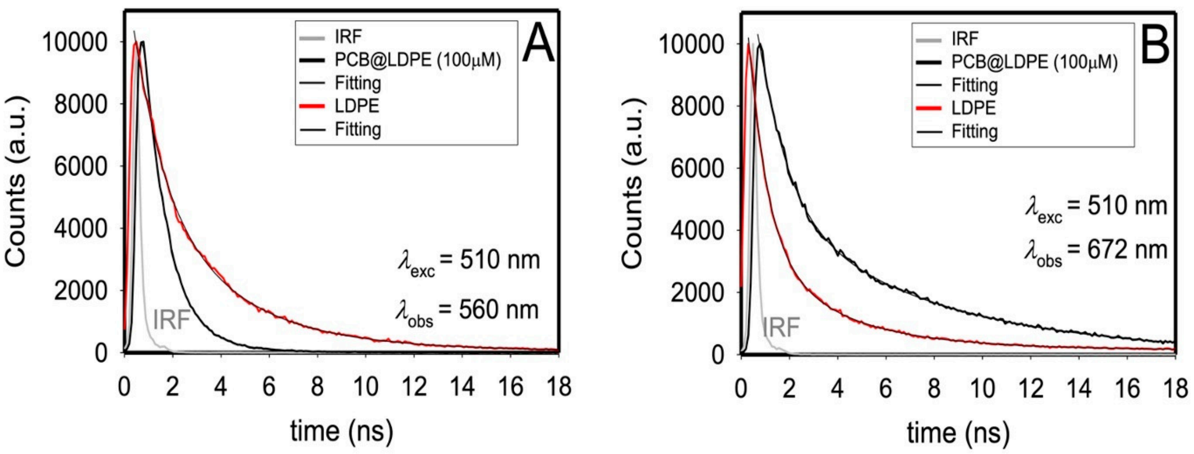

Figure S10. PL decays of solid PCB@LDPE films prepared upon loading PCB dyes at 100 μM at 298 K (black lines). The excitation and monitoring wavelengths are indicated directly in the graph. The data for LDPE (red lines) are added for comparison purposes.

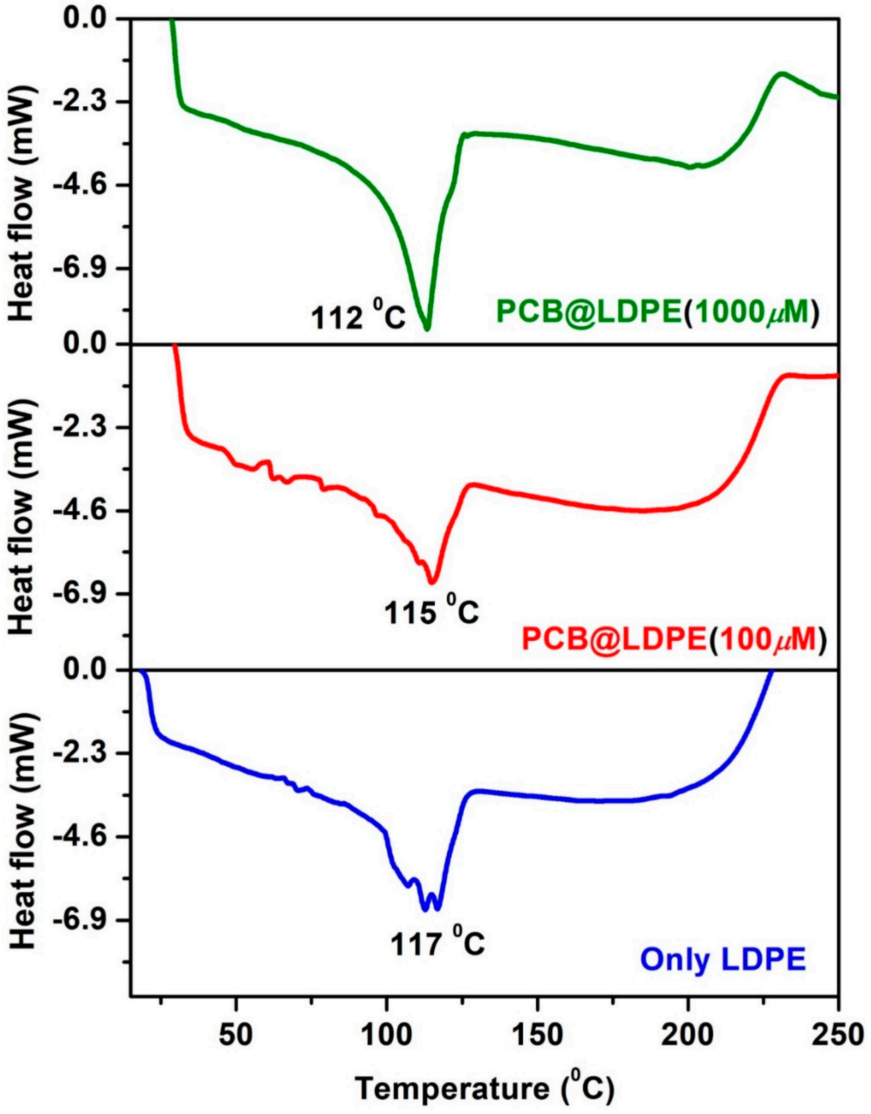

Figure S11. DSC curves of solid PCB@LDPE films prepared upon loading PCB dyes at 100 (red line) and 1000 μM at 298 K (green line). The DSC curve for LDPE (blue line) was added for comparison purposes.

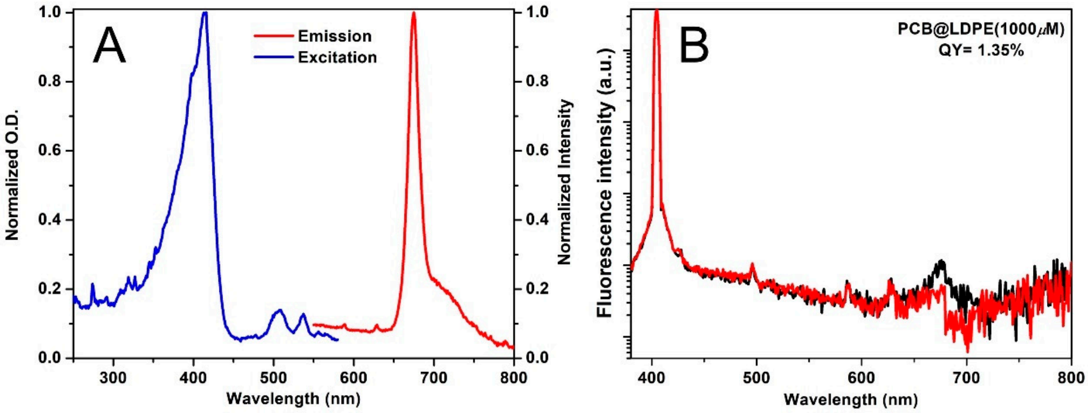

Figure S12. (A) PLE (blue) and PL (red) spectra (λ_{exc} = 400 nm and λ_{mon} = 672 nm) of solid PCB@LDPE films prepared upon loading 7% of PCB dyes at 298 K. (B) the measured Quantum Yield (QY) at 672 nm as directly indicated in the paragraph.

References

1. Hoffmann, E.A.; Fekete, Z.A.; Korugic-Karasz, L.S.; Karasz, F.E.; Wilusz, E. Theoretical and experimental x-ray photoelectron spectroscopy investigation of ion-implanted nafion. *Journal of Polymer Science Part A: Polymer Chemistry* **2004**, *42*, 551-556.
2. Friedman, A.K.; Shi, W.; Losovyj, Y.; Siedle, A.R.; Baker, L.A. Mapping microscale chemical heterogeneity in nafion membranes with x-ray photoelectron spectroscopy. *Journal of The Electrochemical Society* **2018**, *165*, 733-741.
3. Göller, A.H.; Strehlow, D.; Hermann, G. The excited-state chemistry of phycocyanobilin: A semiempirical study. *ChemPhysChem* **2005**, *6*, 1259-1268.
4. AlMaadeed, M.A.; Nógellová, Z.; Mičušík, M.; Novák, I.; Krupa, I. Mechanical, sorption and adhesive properties of composites based on low density polyethylene filled with date palm wood powder. *Materials & Design* **2014**, *53*, 29-37.
